# Association between sella turcica bridging and altered direction of dental eruption: A case-control study

**DOI:** 10.4317/jced.56165

**Published:** 2019-10-01

**Authors:** Ignacio Arcos-Palomino, Josep M Ustrell-Torrent

**Affiliations:** 1 Licenciate in Odontology, University of Barcelona, Orthodontic Clinic Dr. Arcos, Girona, Spain; 2 Full Professor, Director of the Faculty of Odontology, University of Barcelona, Oral Health and Masticatory System Group, Bellvitge Biomedical Research Institute (IDIBELL), L’Hospitalet de Llobregat, Barcelona, Spain

## Abstract

**Background:**

Calcification between the anterior and posterior clinoid processes, also the so-called sella turcica bridging, has been associated in some studies with skeletal anomalies as well as with dental and eruption disturbances. It was hypothesized that sella turcica bridging was associated with an altered direction of dental eruption. The aim of the study was to assess whether there was a relationship between the degree of calcification and the presence or absence of an alteration in the tooth eruption direction.

**Material and Methods:**

A case-control multicenter study was conducted. The study population consisted of 150 subjects (age 10-50 years), 30 of which presented some type of alteration of the direction canine eruption (impactation or transposition) (cases) and 120 selected at random who did not present altered direction of dental eruption (controls). Cases and controls were matched by age, sex, and approximate date of starting orthodontic treatment. Lateral cephalometric radiographies were obtained and the extent of the sella turcica bridging was measured using a Vernier caliper and scored as no calcification, partially calcified, and completely calcified.

**Results:**

The frequency of a partially or completely calcified sella turcica bridge was significantly higher among cases with altered direction of dental eruption as compared with controls. There was a trend towards a shorter length of sella turcica in subjects with two or more canine eruption alterations. Sella turcica bridging was unrelated to sex, but it was significantly influenced by age.

**Conclusions:**

Sella turcica bridging is frequently detected in subjects with altered direction of dental eruption of canines.

** Key words:**Dental eruption, sella turcica bridging, anterior clinoid process calcification, posterior clinoid process calcification, cephalometric radiographs.

## Introduction

Cephalometric measurements are mainly used to assess skeletal and dental patterns for predicting facial growth. Cephalometric analysis is also useful for the diagnosis of other conditions affecting the skull, the face, and the superior cervical spine. Different studies have reported skeletal anomalies and normal variants on cephalometric radiographs seen in orthodontic patients, as well as calcification between the anterior and posterior clinoid processes, also the so-called sella turcica bridging (1). In normal subjects, the prevalence of sella turcica bridging ranges between 3.8% and 13% (2-4). A clear tendency towards a higher occurrence of sella turcica bridge calcification in patients with severe craniofacial deviations (5) as well as in the presence of dental anomalies (6) has been reported.

Development and formation of sella turcica and teeth share in common the involvement of neural crest cells. In fact, it is believe that the anterior part of sella turcica develops mainly from cells of the neural crest, whereas progenitor cells of dental epithelium differentiate by sequential and reciprocal interaction with the neural crest-derived mesenchymal tissue (7). The most frequent alterations of the direction of dental eruption include ectopic eruption of canines with possible canine impactation, and dental transposition (8,9). The most frequent alterations of the direction of dental eruption include ectopic eruption of canines with possible canine impactation, and dental transposition (8,9).

Prevalence of maxillary canine impactation ranges from 1% to 2.5%, being twice as common in females (9). An extensive list of etiologies causing maxillary permanent canine impaction has been reported, including local causes, systemic disorders, and genetic factors. The guidance theory of eruption of the maxillary anterior teeth and the genetic theory have been proposed to explain the etiology of maxillary impacted canine (10-12). The genetic theory is further supported by the association between canine impactation and other genetic anomalies, such as submerged primary molars, enamel hypoplasia, other congenitally missing teeth, or upper lateral incisors microdontia (13,14). Early detection and timely intervention of impacted canines can reduce the time and complexity of permanent dentition treatment. Conventional two-dimensional and three-dimensional images are routinely used to diagnose the position and expected pathway of permanent canine eruption. These radiographs are also a diagnostic tool to detect skeletal variations related to the skull and cervical spine, including the abnormal morphology of the sella turcica.

On the other hand, transposition of teeth has been defined as a form of ectopic eruption which involves a change in the position of two adjacent teeth or the development and eruption of a tooth in a position normally occupied by a non-adjacent tooth (13,15). The etiology of this anomaly remains unclear, although it has been suggested that genetic and environmental factors play a role in this multifactorial condition (16,17). Dental anomalies frequently associated with tooth transpositions include missing lateral incisors and second premolars, undersized lateral incisors, retained deciduous canines, impaction of permanent canines and central incisors, and severely rotated adjacent teeth (13,15). Females have more transpositions than did males, and substantially more transpositions are unilateral, with a moderate left side dominance (13). Retained primary teeth and trauma may also contribute to tooth transposition (17,19).

Recently, sella turcica bridging has been associated with dental anomalies, such as canine ectopic eruption and transposition (5,6,20-23). The presence of intracranial 

clinical calcifications in addition to those affecting teething, strongly suggests a genetic etiology (8). In fact, some studies on the shape of sella turcica have reported significant variations associated with this structure (1). Also, it has been shown that the morphological aspect is established early in the embryonic development (1,24). In the general population, a general prevalence of sella turcica bridging ranging between 1.1% and 13% has been reported (2,3,25), with higher prevalence rates in subjects with severe craniofacial deviations (4,5). However, a distinction between a real bone fusion and an overlapping between the anterior and posterior clinoid processes is difficult to identify (3). Also, sella turcica bridging has been observed in patients with inherited developmental conditions, such as nevoid basal cell carcinoma (NBCCS) and Williams syndrome (3).

The present study was conducted to provide further data on sella turcica bridging in subjects with altered direction of dental eruption.

## Material and Methods

-Study Design and Participants

A prospective case-control study was designed in subjects requiring orthodontic treatment attended in the framework of the Master of Orthodontics at the Dentistry Clinic of the University of Barcelona, as well as in other orthodontic clinics of the researchers located in San Joan Despí, Igualada, and Girona (Catalonia, Spain). The primary objective of the study was to determine the prevalence of sella turcica bridging in subjects with altered direction of dental eruption. The secondary objective was to assess the relationship between sella turcica bridging and demographic characteristics of participants.

The study population consisted of 150 subjects, 67 men and 83 women, aged between 10 and 50 years, 30 of which presented some type of alteration of the direction of dental eruption (premolars, canines) (impactation or transposition) (cases) and 120 selected at random who did not present altered direction of dental eruption (controls). Transposition was defined as any deviation from the normal trajectory leading to location of premolars or canines in a location other than their normal site within the alveolus. Tooth impactation was defined as the infraosseous position of the tooth after the normal expected time of eruption. For inclusion in the study, the case records of all participants were required to include good quality lateral cephalometric radiographs allowing reliability and clear reproduction of measurements of the sella turcica, as well as availability panoramic radiographs to confirm the presence or absence of alteration of dental eruption. The type of malocclusion was not an exclusion criterion. Subjects with severe craniofacial deviations who required combined surgery-orthodontic treatment were excluded from the study. Cases and controls were matched by age, sex, and approximate date of starting orthodontic treatment. The study was approved by the Ethics Committee of the Master of Orthodontics and written informed consent was obtained from all participants.

-Cephalometric Analysis

**Figure 1:**
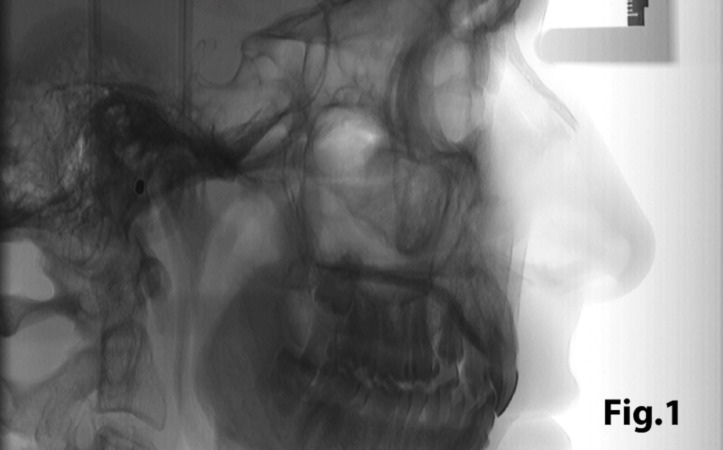
Lateral skull teleradiography with inverting contrast enabling clear visualization of structures.

**Figure 2:**
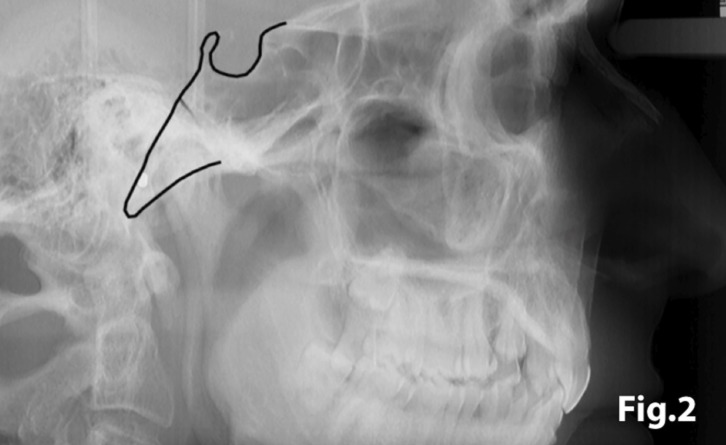
Lateral skull teleradiography showing the tracing of sella turcica.

**Figure 3:**
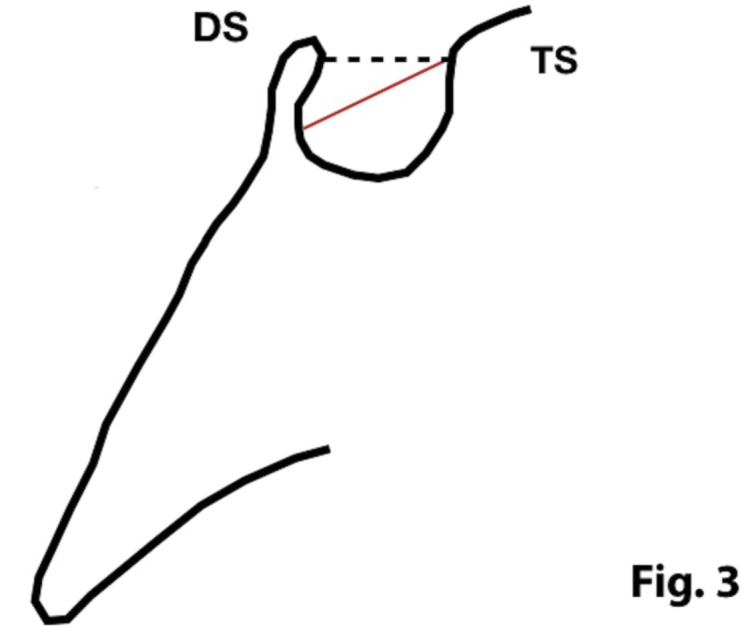
Morphology of sella turcica with reference lines used to measure its size. DS: dorsum sellae; TS: tuberculum sellae. Length (continuous line) and anteroposterior greatest diameter (discontinued line) of the sella turcica.

In order to quantify the extent of a sella turcica bridge from each profile radiograph, the contours of the pituitary fossa were traced from the tip of the dorsum sellae to the tuberculum sella (Figs. 1,2). The length of the sella turcica was measured as the distance from the tuberculum sella to the tip of the dorsum sellae, and the anteroposterior greatest diameter was measured from the tuberculum sella to the most posterior point on the inner wall of the pituitary fossa (Fig. 3), using a permanent 0.5-mm lead pencil with the aid of a negatoscope in a dark room. Measurements were taken by one of the authors (I.A.P.). Duplicate tracings were made by the same author on 20 films on two separate occasions with a 15-day interval between tracings to assess the random error. The measurement errors ranged from 0.12 to 0.18 mm for both length and diameter and were not significant. On this basis, it was considered that the experimental error was unlikely to bias the accuracy of the sella measurements.

**Figure 4:**
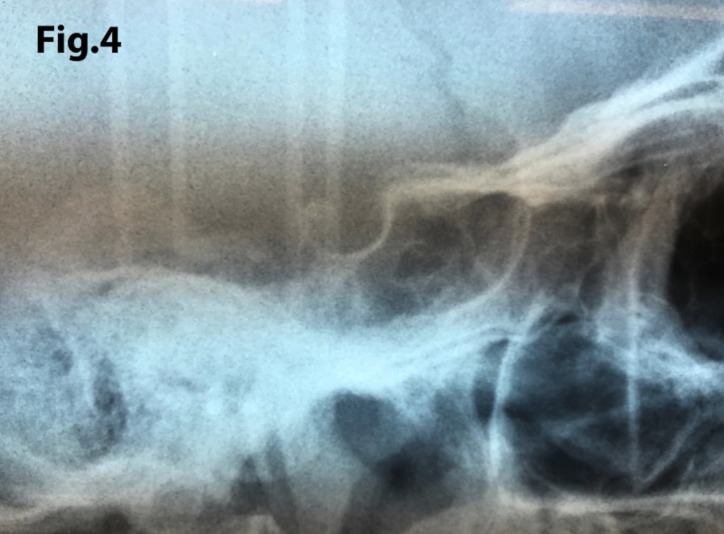
A case of sella turcica scored as type I (no calcification). The length is greater than or equal to three-fourths of the anteroposterior diameter.

**Figure 5:**
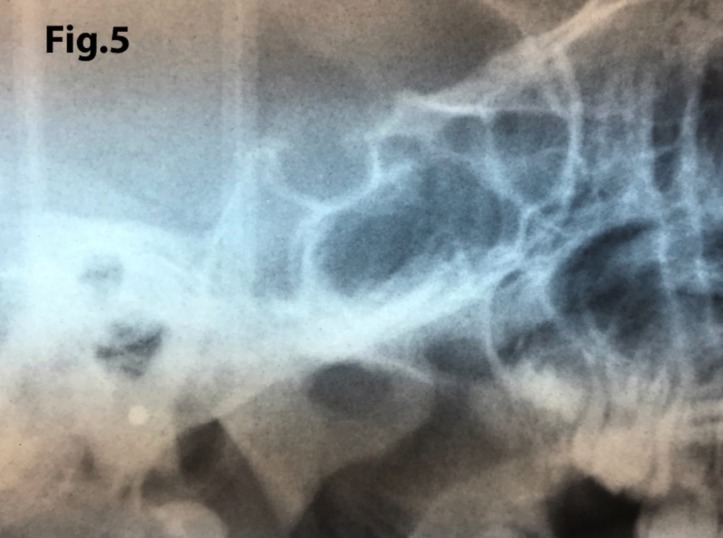
A case of turcica scored as type I (no calcification). The length is greater than or equal to three-fourths of the anteroposterior diameter.

**Figure 6:**
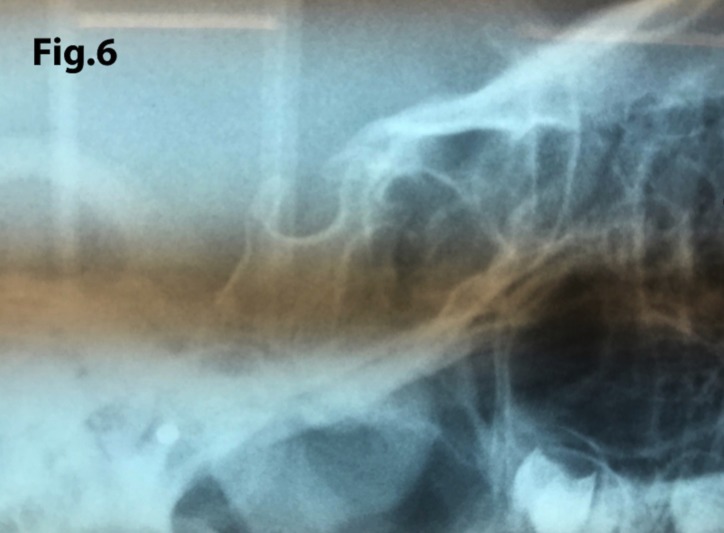
A case showing a partially calcified bridge (type II) with length of sella turcica less or equal to three-quarters of the anteroposterior diameter.

**Figure 7:**
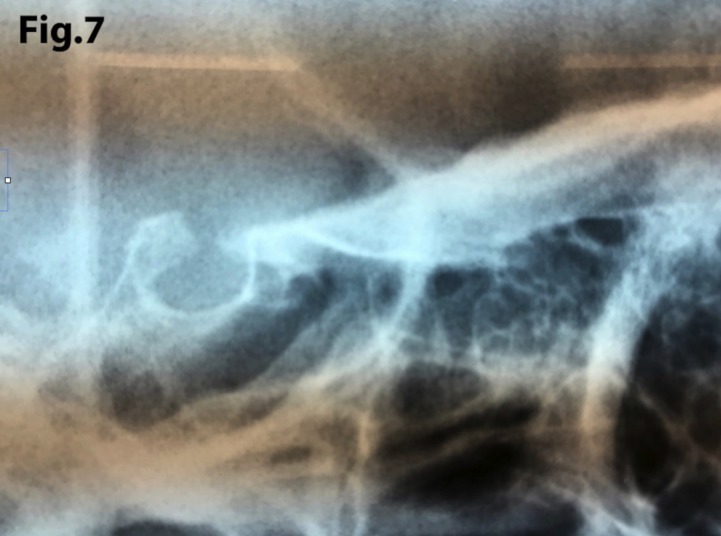
Similar findings than in Figure 6 but with a shorter length of sella turcica indicating that there is greater calcification (type II).

**Figure 8:**
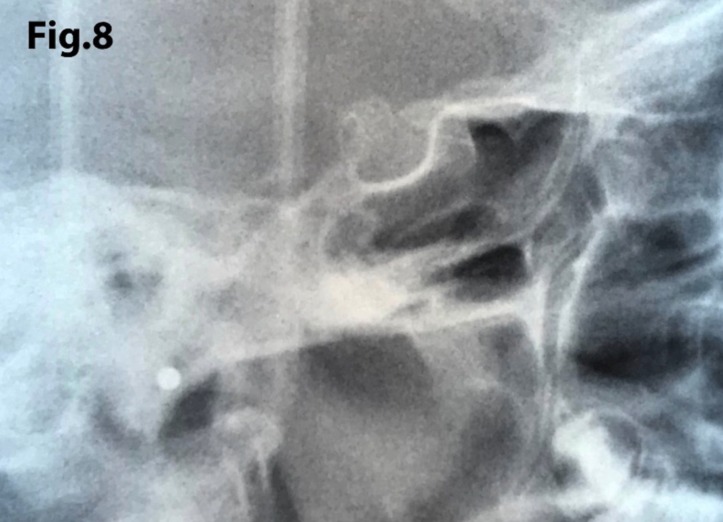
Sella turcica bridging (type III). The length is ≤ 0.1 mm.

**Figure 9:**
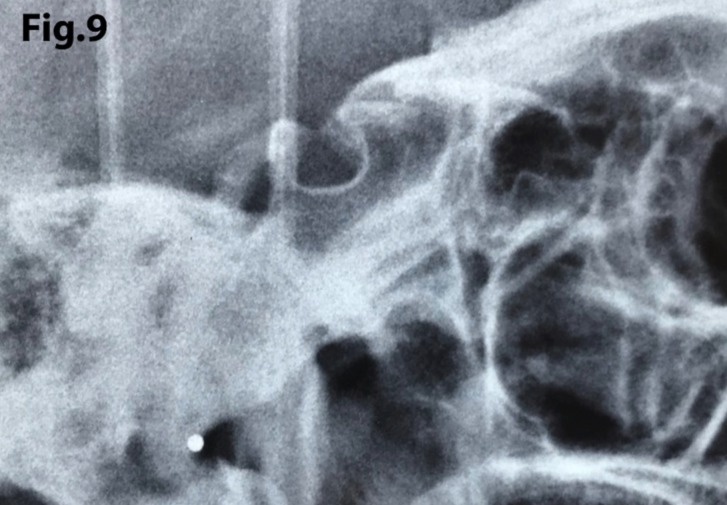
A case illustrating type III calcification with sella turcica bridging.

The length of sella turcica was considered representative of the bridge calcification. A Vernier caliper was used for measurement of the extent of sella turcica bridging and scored according to a standardized scoring scale, which was established by comparing of the sella turcica length and diameter. If the length of the sella turcica was greater than or equal to three-fourths of the diameter, the sella was scored as type I (no calcification) (Figs. 4,5); if less than or equal to three-quarters (bridge partially calcified) as type II (Figs. 6,7); and type III for a radiographically visible diaphragma sellae (bridge completely calcified) (Figs. 8,9). For the purpose of analysis, the three types of calcification were recodified as a categorical variable of presence of calcification (type II or III) versus absence of calcification (type I).

-Statistical Analysis

Categorical variables are expressed as frequencies and percentages. The Kolmogorov-Smirnov test was used to assess the normal distribution of continuous variables. Continuous variables are expressed as median and interquartile range (25th-75th percentile). The distribution of variables between cases and controls was analyzed using the chi-square (χ2) test for categorical variables, and the Mann-Whitney U test or the Kruskal-Wallis test for the comparison of 2 or 3 or more groups of continuous data, respectively. Statistical significance was set at *P* < 0.05. Logistic regression analysis was used to assess the strength of the association between calcification and the study group. Crude and adjusted odds ratio (OR) by demographic variables and the corresponding 95% confidence intervals (CIs) were calculated. The Statistical Package for Social Science (SPSS) version 21.0 was used for data analysis.

## Results

Alterations of the direction canines eruption was diagnosed in 30 of the total 150 study subjects, with a prevalence rate of 20%. In these subjects, a total of 39 alterations were observed. Twenty subjects had 1 alteration and the remaining 28 had 2 or more alterations. The type of alteration was an impacted canine in 28 subjects, canine transposition in 1, and both impactation and transposition in 1. Most alterations (n = 25) were found in the maxilla (right hemimaxilla n = 13, left hemimaxilla, n = 7, both sides, n = 5). There was only one case of altered direction of dental eruption in the mandible, whereas in four subjects, alterations affected both the maxilla and the mandible.

There were 67 men and 83 women, with a median age of 16 years (range 13-23). The distribution of cases (n = 30) and controls (n = 120) according to demographic variables, and length and anteroposterior diameter of the sella turcica is shown in  Table 1. The percentages of men and women were similar among cases and controls, but controls were significantly younger than cases. The length of sella turcica was shorter in cases, whereas the anteroposterior diameter was greater in cases than in controls.

**Table 1:**
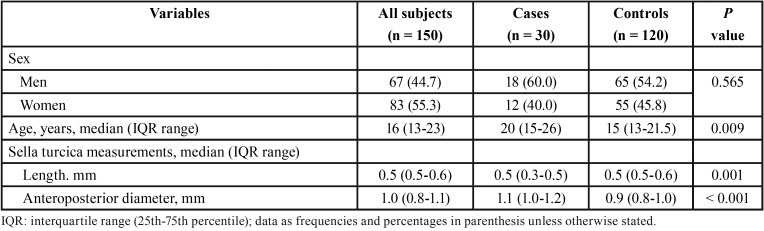
Demographic characteristics and measurements of the sella turcica in cases and controls.

The distribution of the number of alterations of eruption direction according to demographic variables and measurements of the sella turcica is shown in  Table 2. IN the group of subjects with 1 alteration, differences between men and women were not observed, but most cases with 2 or more alterations were women. Length and anteroposterior diameter of the sella turcica were also similar, although there was a trend of a shorter length in the presence of 2 or more dental eruption alterations. Also, the single case of mandibular altered direction of dental eruption showed a shorter length as compared with alterations found in the maxilla, and the 4 cases of maxillary/mandibular alterations showed a trend towards a longer length ( Table 3).

**Table 2:**
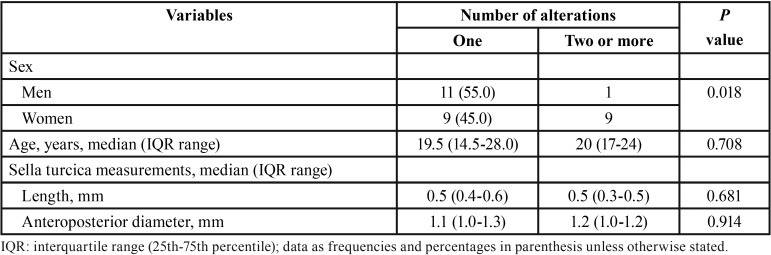
Demographic characteristics and measurements of the sella turcica according to the number of alterations of direction of dental eruption.

**Table 3:**
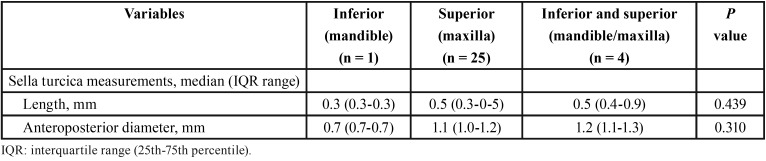
Demographic characteristics and measurements of the sella turcica according to location of alterations of direction of dental eruption.

Of the total 150 study subjects, there were 78 (52%) subjects without calcifications. In the remaining 72 subjects, type II calcifications were documented in 69 and type III in 3. As shown in  Table 4, sella turcica bridging was absent in 59.2% of controls, whereas sella turcica bridging was present in 76.6% of cases. Also, the percentage of calcification was greater in subjects with altered direction of dental eruption (76.7%) than in subjects without alterations (40.8%). The presence of sella turcica bridging was unrelated to sex, but subjects with bridging were a mean of 2.5 years older than controls. In the multivariate analysis, the probability of sella turcica bridging was 4.76 times greater in cases than in controls (OR 4.76, 95% CI 1.90-11.96) and 4.34 times greater when adjusted by age (OR 4.34, 95% CI 1.71-11.03) ( Table 4). However, an association between the presence of sella turcica bridging and the number and location of altered direction of dental eruption was not found ( Table 5).

**Table 4:**
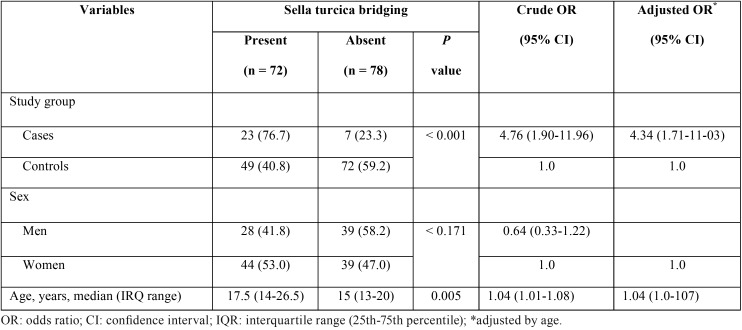
Sella turcica bridging in the study groups according to demographic characteristics. Results of multivariate analysis.

**Table 5:**
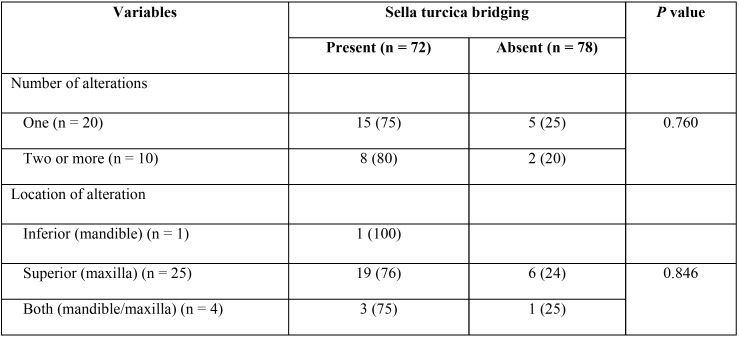
Sella turcica bridging and characteristics of alterations of dental eruption direction.

## Discussion

In the present study, we assessed the frequency of sella turcica bridging in a sample os subjects presenting for orthodontic treatment because of altered direction of eruption of the canines. Rational of the study was supported in findings of previous studies published in the literature showing a higher prevalence of altered direction of dental eruption in association with sella turcica bridging (5,6,20-23). Also, it has been reported that dental anomalies associated with sella turcica bridging may be a characteristic feature of some inherited developmental craniofacial syndromes (3). The presence of a partially or completely calcified bridge of the sella turcica in subjects with altered direction of dental eruption, currently provides a genetic-based further evidence for subjects affected of these conditions. As sella turcica bridging occurs early during the developmental period, it has been suggested as a screening test for possible genetic diseases and dental alterations (25). Certainly, the findings of this study indicate that subjects with sella turcica bridging have a potential risk of developing alterations of dental eruption direction. However, the diagnosis of sella turcica bridging should be establish with caution since radiographic overlapping of the anterior and posterior clinoid processes may be confused with actual bone fusion (3,5).

Although most cases of sella turcica bridging are detected at early stages in life, in some cases, calcification develops over time and could only be observed on lateral cephalometric radiographs taken in a later stage. In the present study, it was found that sella turcica bridging was influenced by age. In fact, the older age, the higher probability of bridging (1.04-fold increase per year). In the logistic regression analysis, the influence of age on sella turcica bridging was maintained in the logistic regression model adjusted for age and by study group. Other studies have also found a correlation between sella turcica bridging and age. In a study of northern Italian patients 300, in which 300 computed tomography scans of the head were revised, the prevalence of sella turcica bridging was 16% (25). Significant differences according to sex were not found, which is in agreement with our study, but a correlation with age was observed (*P* = 0.007). In the study of 34 adolescents with a palatally displaced canine and second mandibular premolar aplasia, age was a predictor that significantly explained the degree of calcification of the sella turcica (6).

While prevention with early diagnosis of such an alteration could potentially improve management of dental eruption disorders especially cases of tooth impactation and transposition, we must be careful because the only way to diagnose early sella turcica bridging is with a lateral skull radiograph, which following current guidelines is only justified in children under 10 years, when there are serious skeletal discrepancies, which require early treatment. However, results of the present study may suggest that, in some cases, particularly in cases of family history of dental impaction or transposition, the radiographic examination of the sella turcica could be considered as an additional predictor. This could allow the application of adequate measures to prevent subsequent dental impaction or transposition.

In summary, subjects with altered direction of eruption of canines showed a higher occurrence of sella turcica bridging than controls, with a trend towards a shorter length of sella turcica in subjects with two or more canine eruption alterations. Sella turcica bridging was unrelated to sex, but it was significantly influenced by age. The diagnosis of sella turcica bridging at an early age may alert clinicians to the possibility of dental eruption alterations, which is relevant since timing for preventive treatment of impacted canine is essential for successful orthodontic treatment outcome.
